# Is There a Relationship between Sperm DNA Fragmentation and Intra-Uterine Insemination Outcome in Couples with Unexplained or Mild Male Infertility? Results from the ID-Trial

**DOI:** 10.3390/life13010011

**Published:** 2022-12-20

**Authors:** Alessa Sugihara, Usha Punjabi, Ella Roelant, Diane De Neubourg

**Affiliations:** 1 Centre for Reproductive Medicine, Algemeen Ziekenhuis Klina, 2930 Brasschaat, Belgium; 2 Centre for Reproductive Medicine, Antwerp University Hospital, 2650 Edegem, Belgium; 3 Department of Reproductive Medicine, Antwerp Surgical Training, Anatomy and Research Centre (ASTARC), Faculty of Medicine and Health Sciences, University of Antwerp, 2000 Antwerp, Belgium; 4 Clinical Trial Centre (CTC), CRC Antwerp, Antwerp University Hospital, University of Antwerp, Drie Eikenstraat 655, 2650 Edegem, Belgium

**Keywords:** sperm DNA fragmentation, insemination, male infertility

## Abstract

Background: Sperm DNA fragmentation has been proposed as a candidate test for the assessment of sperm function on the premise that damage to the sperm chromatin is associated with a detrimental reproductive outcome. The objective of our study was to investigate whether sperm DNA fragmentation testing has a prognostic value, and thus can play a pivotal role in selecting future patients for intra-uterine insemination (IUI) therapy. Methods: This was a prospective cohort study conducted in a University Hospital setting. SDF was measured through TUNEL assay on the fresh semen sample presented at diagnosis and at insemination in couples with idiopathic/mild male infertility undergoing natural cycle IUI treatment. The generalized estimating equation (GEE)-model and multivariable model were used to analyze the probability of live birth and clinical pregnancy, respectively. ROC analysis was carried out to determine an SDF cut-off. Results: There was an inverse relationship between SDF in the ejaculate of the diagnostic semen sample and CP (*p* = 0.02; OR 0.94 95% CI (0.90, 0.989)) as well as LB (*p* = 0.04; OR 0.95 95% CI (0.90, 0.9985)). No significant association was found between SDF after gradient and IUI outcome in the diagnostic sample nor between SDF (ejaculate/after gradient) in the IUI samples. The ROC analysis proposed a cutoff of 17.5% as the best compromise between sensitivity and specificity in the diagnostic SDF for live birth; however, the test diagnostics are low, with an AUC of 0.576. Conclusions: Overall, this study strengthens the hypothesis of an inverse relationship between SDF and CP/LB. Furthermore, SDF taken together with other clinical characteristics might provide more insight into male reproductive potential and predicting IUI outcome. Couples with SDF ≥ 17.5% in the diagnostic semen sample did not reach live birth. Further research is necessary to establish the diagnostic and prognostic potential of SDF as an add-on test.

## 1. Introduction

To date, much uncertainty exists about the relevance of semen parameters in predicting male reproductive potential [1], leaving both patients and clinicians in the dark. The golden standard of the male fertility work-up is semen analysis performed according to the WHO manual [2], which provides valuable information in cases with a severe male factor. However, its relevance for the choice of treatment and its predictive value for infertile couples with idiopathic or mild male infertility embarking on medically assisted reproduction (MAR) remains disputed [3]. Sperm DNA fragmentation (SDF), that is, damage in the male germ line in the form of single- or double-strand breaks occurring at testicular, epididymal, or post-ejaculatory levels, has gained a considerable amount of interest over the years based on the premise that damage to the sperm chromatin is associated with a detrimental reproductive outcome [4,5,6,7], and thus has been proposed as a candidate test for the assessment of sperm function. The integrity of the genetic information contained within the sperm cells is continuously challenged by both intrinsic factors (protamine deficiency, excess reactive oxygen species (ROS) levels, apoptosis) and extrinsic factors (e.g., testicular hyperthermia, environmental toxins), resulting in different degrees of DNA damage [8]. Although a certain degree of DNA fragmentation is inherent to the process of chromatin compaction, high levels of SDF have been linked to lower fertilizing potential of the sperm [9], lower clinical pregnancy numbers [10] and higher risk of miscarriage [7]. Interestingly, a significant number of subfertile men have been reported to have high levels of sperm DNA damage despite normal semen parameters [4,11]. Nonetheless, the evidence pertaining to SDF and fertility outcomes remains conflicted [12,13].

The vast majority of studies on SDF have focused on IVF/ICSI outcome [12,13,14,15,16], with numerous papers suggesting ICSI [17] or even ICSI/TESE [18,19,20] as the preferred treatment for couples with infertility and high sperm DNA fragmentation [21]. This notwithstanding, we believe it is important to take a step back and to establish the evidence of SDF testing with regard to pregnancy outcome after intra-uterine insemination (IUI). IUI represents a simple and noninvasive first-line treatment, and is pointed out as being one of the most frequently used fertility treatments on the worldwide scale [22,23]. A systematic review of three studies by Zini in 2011 [24] concluded that sperm DNA damage is associated with lower IUI pregnancy rates. Our recent updated review of eight studies failed to bring forward a clear recommendation for clinical practice due to conflicting results [25,26,27,28].

Interestingly, there are no reports on longitudinal data concerning sperm DNA fragmentation and IUI outcome, even though couples often go through more than one IUI cycle before obtaining a positive result. Moreover, the available literature on this topic has mostly concentrated on SDF in the IUI sample, which while it has merit in terms of the scientific knowledge on the topic, has a limited clinical role, as caregivers wish to estimate the couple’s chance of success before assigning them to the IUI treatment arm. The psychological burden of treatment [29] as well as the socio-economic consequences of unsuccessful fertility treatments [30] urge improvement in the efficacy of treatment plans and better management of patient expectations. The objective of our present study was to investigate whether SDF testing in a longitudinal IUI trial has prognostic value, and thus can play a pivotal role in selecting future patients for IUI therapy. This paper outlines the applied materials and methods, shares the results from couples with idiopathic or mild male infertility undergoing up to four IUI cycles, and presents these results in the context of the current literature.

## 2. Materials and Methods

### 2.1. Study Design and Participants

This was a monocentric prospective cohort study conducted at the Centre for Reproductive Medicine of the Antwerp University Hospital, Belgium (clinicaltrials.gov NCT03319654). All couples underwent a medical and fertility history taking followed by basic fertility investigations such as semen analysis, pelvic ultrasound, and tubal patency testing (Chlamydia antibody test, hysterosalpingography (HSG)/hysterosalpingofoamsonography (HyfoSy) or laparoscopy). During the period of October 2017 to October 2020, couples who were about to initiate a natural cycle IUI treatment protocol after an infertility duration of minimally 12 months were invited to join the study. As such, only women without a discernible ovulation disorder/grade III or IV endometriosis/double-sided tubal disease and men without a severe male factor were invited to join the study, which was in an attempt to obtain as homogenous a population as possible.

Female partners were between 18 and 40 years of age with reported regular menstrual cycles (defined as 24 to 38 days) [31], with a body mass index (BMI) ranging between 18–30 and confirmed patency of at least one fallopian tube on HSG/Hyfosy and/or laparoscopy, with a normal uterine cavity on either ultrasound, HSG/Hyfosy, or hystero-/laparoscopy. 

Male partners were between 18 and 65 years of age, with a BMI ranging between 18–30, with a normal semen analysis or mild male subfertility.

Couples were classified as having unexplained subfertility when the fertility investigations showed at least one patent fallopian tube, a regular ovulatory menstrual cycle, and a normal semen analysis. Mild male infertility was defined as one or more abnormal semen parameters with a pre-wash total progressive motile sperm count above 5 million according to the WHO 2010 manual [2].

The couples underwent natural cycle IUI with or without ovulation trigger until pregnancy was achieved for a maximum of four cycles.

Informed consent to use the sperm rest fractions for DNA fragmentation testing was obtained from all the participants; in addition, the patients provided their consent to use their clinical data for research purposes. In case of a confirmed pregnancy, follow-up was carried out until delivery in line with the national Belgian ART registration policy [32]. Patients were classified as lost to follow-up in the case that they did not return to start treatment within 6 months of inclusion. The study protocol was approved by the Ethical Committee of Antwerp University hospital and the University of Antwerp (Belgian registration number B300201733352, approved on 11 September 2017). This paper was written in compliance with the STROBE recommendations [33].

### 2.2. Treatment Protocol

The women underwent an ultrasonography and venous blood sampling (LH, estradiol, progesterone) in a natural cycle on day 10–12; this was repeated approximately every other day until a dominant follicle of 17 mm or more was measured. In case the menstrual cycle proved to be prolonged with the absence of a growing follicle ≥12 mm on cycle day 17–20, clomiphene citrate (CC) 50 mg was given during 5 days. In case the patient returned for a subsequent cycle, CC was started on cycle day 3. In the absence of an LH surge, 250 µg hCG or 5000 IU (Ovitrelle^®^,Merck nv, Overijse, Belgium or Pregnyl^®^ MSD Belgium, Brussels, Belgium) was administered to induce ovulation when a maximum of two dominant follicles were present. The IUI was performed between 32–40 h after hCG administration or ±24–26 h after detection of the spontaneous LH surge. The capacitated motile sperm was inseminated by a gynecologist in the uterine cavity.

### 2.3. Semen Analysis

The semen analysis and preparation were performed as previously described by Punjabi 2018 et al. Patients were instructed to maintain 2–7 days of sexual abstinence. All semen samples were collected at the laboratory, and any missing ejaculate fraction was reported. Samples were weighed for volume and analysis was initiated within 60 min after ejaculation, including sperm concentration, using an improved Neubauer hemocytometer (Marienfeld GmbH, Lauda-Königshofen, Germany) combined with a positive displacement pipette (Microman, Gilson Inc., Middleton, WI, USA); sperm motility, including progressive and total motile sperms; and sperm morphology, adapting the modified Papanicolaou stain (Sigma-Aldrich Inc., St. Louis, MO, USA).

### 2.4. Sperm Processing

A part of the same semen sample was treated with a two-step discontinuous density gradient [22] using PureSperm^®^ (Nidacon, International AB, Gothenburg, Sweden). Briefly, 40% and 80% PureSperm^®^ density gradients were prepared using 1.5 mL of each suspension. Semen (1.0–1.5 mL) was layered on the top of each gradient and centrifuged for 20 min at 300× *g*, after which the upper layer seminal plasma, the 40% upper layer, and the 40–80% interface were discarded and the remaining spermatozoa in the 80% pellet were collected from the bottom of the tube and washed once for 10 min with human tubal fluid (HTF Hepes, Gynotec, Malden, The Netherlands) supplemented with albumin (human albumin 20%, CAF-DCF, Brussels, Belgium).

### 2.5. SDF Testing

The TUNEL assay was performed on a sperm fraction of the diagnosis and IUI samples, as previously described by Punjabi et al. 2019. The spermatozoa were incubated for 30 min at 37 °C with LIVE/DEAD^®^ Fixable Dead Cell Stain (far red) (Molecular Probes, Life technologies, Eugene, OR, USA), after which the cells were washed 2× with phosphate-buffered saline (PBS, GIBCO Life technologies, Paisley, UK) before being incubated with 2 mM dithiothreitol (DTT, Sigma-Aldrich, Belgium) for 45 min. Following this, the samples were washed 2× in PBS and fixed in 3.7% formaldehyde (Sigma-Aldrich, Bornem, Belgium) for 20 min at 4 °C. As we have previously shown that storage of the sample at 4 °C affects reproducibility [10], the assay was carried out directly after fixation without storage in 0.1 M glycine. For the assay, the spermatozoa were washed 2× and centrifuged before being resuspended in 500 μL of fresh permeabilization solution (100 mg sodium citrate, 100 μL Triton X–100 in 100 mL dH_2_O) and incubated for 5 min at 4 °C. The cells were washed 2× with PBS. The positive control samples were treated with 5 μL of DNase I (Qiagen, Hilden, Germany) 1500 Kunitz Units for 30 min at room temperature. The assay was performed using a Fluorescein In Situ Cell Death Detection Kit (Roche Diagnostics, Mannheim, Germany) with an Accuri C6 flow cytometer (BD Sciences, Erembodegem, Belgium). For each sample, 5000–10,000 events were recorded at a flow rate of 35 μL/min. The test was performed on the ejaculate and after density gradient centrifugation. DNA fragmentation was analyzed, with the results presented as follows: Total SDF: percentage of the entire sperm population that was positive for DNA fragmentation; Vital SDF: percentage of the entire sperm population that was alive and positive for DNA fragmentation. The setup of the study included an SDF analysis in the first three IUI cycles; due to the burden and cost of the analyses, a fourth SDF analysis was carried out only occasionally. 

### 2.6. Intra-Uterine Insemination

On the day of insemination, a semen sample was obtained and analyzed according to the World Health Organization 2010 guidelines [2] and processed by density gradient centrifugation. In case the semen sample was deemed sufficient for IUI with an inseminating progressive motile count (IMC) of ≥2 million [34], the sperm rest fraction was used for immediate SDF testing. The prepared sample was kept at room temperature and insemination was carried out within 120 min after ejaculation using a soft IUI catheter (Wallace^®^ Intrauterine Insemination Catheters, Cooper Surgical, The Hague, The Netherlands) rinsed with HTF and albumin. The inseminating volume was held constant between 0.3 and 0.5 mL.

### 2.7. Treatment Outcome

A clinical pregnancy (CP) with fetal heartbeat was diagnosed by ultrasonography. A miscarriage was defined as the spontaneous loss of an intra-uterine pregnancy prior to 22 completed weeks of gestational age. Live births were registered in case of a birth occurring after 22 completed weeks of gestational age with evidence of life [35].

### 2.8. Statistical Analysis

A power analysis was performed to estimate the sample size needed for the receiver operating characteristics (ROC) curve analysis. Because the literature on the subject of sperm DNA fragmentation as measured by TUNEL flow cytometry and IUI outcome was scarce, there were no reference values available to estimate the area under the curve (AUC) or distribution of live births versus no live births (kappa). In order to detect an alternative AUC varying between 0.7–0.75 compared to a null AUC of 0.5, with a significance level of 5% and power of 90% and a kappa varying between 1:1 and 1:3, we estimated that a minimum of 52 patients and a maximum of 111 patients had to be included. 

Descriptive statistics were calculated for the baseline demographics and IUI characteristics. Continuous variables such as age, duration of infertility, Body Mass Index (BMI), and semen parameters (including SDF) are reported as mean (standard deviation (SD)) and in case of skewness as median (interquartile range (IQR)). Categorical variables such as primary or secondary infertility, smoking, and natural or CC-cycle are reported as absolute numbers (percentages). The small number of missing values can be derived from the tables with the number of available observations. In the GEE model, all available measurements per couple were used.

The baseline and diagnostic semen characteristics are compared between the couples who reached live birth in any of the observed cycles and the couples who did not reach live birth in any of the observed cycles using an independent samples T-test (or Mann–Whitney test, as appropriate) for the continuous variables and Chi-square test (or Fisher’s exact test, as appropriate) for the categorical variables.

A generalized estimating equation (GEE) model with an exchangeable correlation structure and a logit link function was used to analyze the probability of live birth and clinical pregnancy, respectively. All IUI cycles are used in this model, with correction for the fact that cycles of the same couple are not independent. First, a univariate analysis was considered including only one variable as a predictor in the model to evaluate the association between this variable and the outcome. Second, a multivariable model was considered, including the variables found significant in the univariate analysis; a 10% significance threshold was used. Finally, a multivariable analysis including one of the SDF variables together with a baseline patient or semen characteristic was considered to evaluate the association of the SDF with the outcome after correction for these variables. 

An ROC analysis was performed using the total SDF in the ejaculate in the diagnosis sample to discriminate between live birth in any of the cycles or no live birth in any of the cycles. A cut-off based on the Youden index was used to calculate sensitivity, specificity, and positive and negative predictive value. For this, live birth was defined as a ‘case’ for which a low SDF value was an indicator. The predicted probabilities of logistic regression models with the total SDF in the ejaculate in the diagnosis sample, female age, male smoking, and male BMI as predictors were considered. Sensitivity and specificity were calculated for different cut-offs taken from the literature. Finally, the predicted probabilities of live birth (and clinical pregnancy) from the GEE model using the total SDF in ejaculate in the diagnosis sample, male BMI, and smoking status on the one hand and total SDF in the ejaculate in the diagnosis sample, male BMI, and male age on the other hand were used in an ROC analysis.

All analyses were conducted using R 4.1.0.

## 3. Results

**Figure 1:**
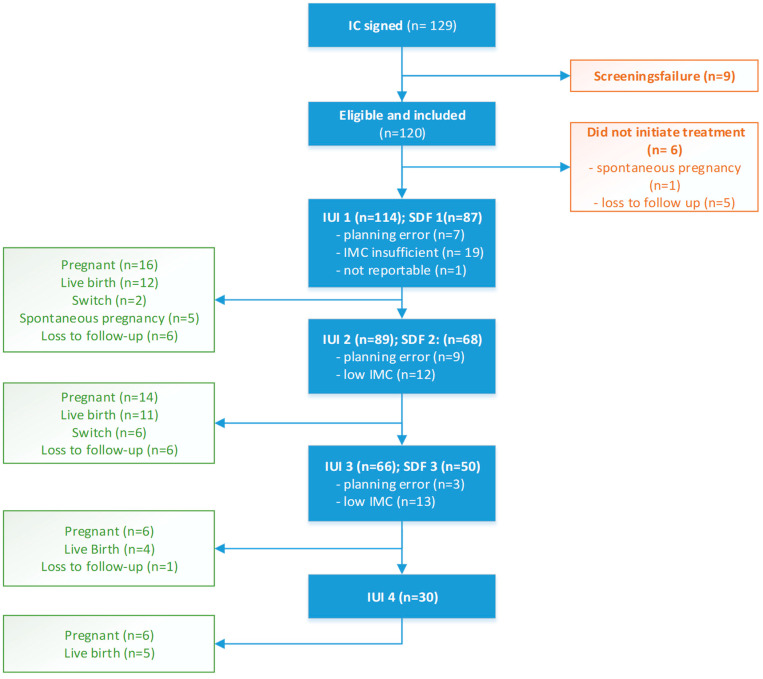
Patient inclusion and treatment flowchart.

In all, 120 patients were included in the study, of whom 114 actually initiated an insemination treatment; for more details, refer to Figure 1. 

The baseline characteristics of the couples are shown in Table 1, while the semen parameters according to the WHO manual along with the SDF values are depicted in Table 2. The median (IQR) SDF total in the ejaculate as measured by TUNEL flow cytometry was 9 (7.2)%. 

### 3.1. IUI Outcome

There were 25 couples (21.9%) with one IUI cycle, 23 (20.2%) with two cycles, 36 (31.6%) with three cycles, and 30 (26.3%) with four cycles. Among the 114 included couples who initiated a natural cycle IUI treatment plan, 41 (36%) couples reached clinical pregnancy and 32 (28.1%) had a live birth. The IUI treatment characteristics are shown in Table 3.

**Figure 2:**
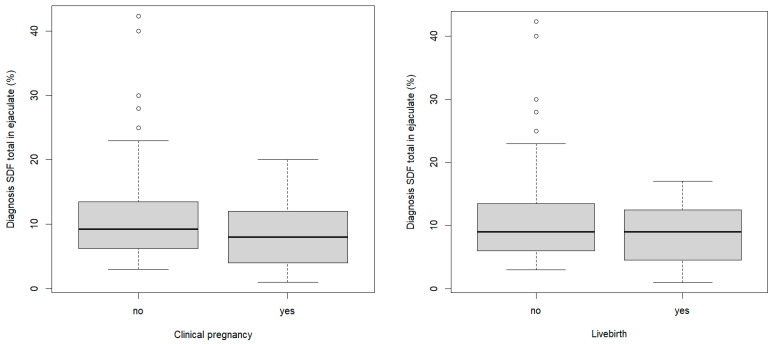
Boxplot SDF total in ejaculate (diagnostic sample) with regards to clinical pregnancy and live birth outcome.

A significant difference in the Hunault score was observed in the patients who reached LB in any of the cycles (15.4 (4.9) versus 19.1 (5.4); *p* = 0.001, T-test). Except for infertility duration (18.2 (7.5) versus 25.3 (13.4) months, *p* = 0.0009, Mann–Whitney U test), there were no significant differences in the baseline characteristics or semen parameters between the couples who reached live birth and those who did not. Figure 2 illustrates the SDF (total in ejaculate) of the diagnostic sample for clinical pregnancy and live birth.

### 3.2. Uni- and Multivariable Analysis

#### 3.2.1. Clinical Pregnancy

Based on our univariate analysis of the probability of clinical pregnancy with the GEE model, a significant effect of SDF diagnosis (*p* = 0.02; OR 0.94 95% CI (0.90, 0.989)) and female age (*p* = 0.03; OR 0.92 95% CI (0.85, 0.99)) can be noted *(*Table 4*).* When combining these significant findings in a multiple variable analysis, the odds in favor of clinical pregnancy decrease by 8% per year of increase in female age when SDF diagnosis remains constant (OR 0.92 (0.95, 0.998); *p* = 0.044), and the odds decrease by 5% per unit increase in SDF when keeping female age constant (OR 0.95 (0.91, 0.994); *p* = 0.026).

#### 3.2.2. Live Birth

The GEE model with live birth as outcome shows a significant effect of total SDF diagnosis in ejaculate and infertility duration (Table 5). A unit increase in infertility duration decreases the odds on live birth by 6% (OR 0.94 95%CI (0.88, 0.993)), and a unit increase in the aforementioned SDF decreases the odds on live birth with 5% (OR 0.95 95% CI (0.90, 0.9985)).When combining the significant findings (infertility duration, mean cycle length and diagnosis SDF) in a multiple variable analysis while keeping the other variables constant, the odds of live birth decrease by 6% per year of increase in infertility duration (OR 0.94 (0.88, 1.002); *p* = 0.06), the odds increase by 20% per unit increase in mean cycle length (OR 1.2 (1.03, 1.40); *p* = 0.02), and finally, the odds decrease by 6% (OR 0.94 (0.89, 0.987); *p* = 0.02) per unit increase in SDF diagnosis. The treatment protocol (clomid versus natural cycle) was not found to be significant (*p* = 0.31).

The multivariable analysis evaluating the effect of SDF variables while correcting for possible confounding factors with one extra variable at a time provides similar results on the effects of the SDF variables.

There were no significant associations (*p* > 0.05) between the other SDF values (SDF total in gradient, SDF vital in ejaculate and in gradient) and either clinical pregnancy or live birth. The same can was the case for the SDF values of the IUI samples. However, the effect of total SDF in ejaculate and after gradient of the IUI sample (*p* = 0.17, *p* = 0.10) were closer to significance when correcting for possible confounders such as mean cycle length.

### 3.3. SDF Cut-Off

ROC curve analysis was carried out and cut-offs were estimated based on the Youden index. The proposed cut-off for SDF total in ejaculate and live birth is 17.5%. The test diagnostics for SDF and live birth reveal a high sensitivity of 100%, very low specificity of 16%, PPV of 30%, and NPV of 100%. Logically, the corresponding AUC is low, at 0.576. When considering the age of the woman and the smoking status and BMI of the man, there is a slight increase in AUC to 0.688. 

When applying the proposed cut-off SDF values from the literature to this study population, specifically, 13% as proposed by Punjabi et al. [36] and 10% by Simon et al. [37], we notice a slight improvement in specificity (25% and 42%, respectively) at the cost of a large decline in sensitivity (75% and 68%, respectively).

Furthermore, we calculated the AUC with the predicted probabilities of live birth using the coefficients from the GEE model. When using a cut-off of 10.6% or 9.5%, the model with SDF, male BMI, and male smoking status and the model with SDF, male BMI, and male age provide a more acceptable AUC(0.68 and 0.65, respectively), sensitivity (73% and 82%, respectively), and specificity (68% and 58%, respectively).

## 4. Discussion

As highlighted in a systematic review from 2019 [8], peer-reviewed studies looking at the application of SDF in IUI settings are scarce. To the best of our knowledge, this is the first prospective longitudinal study evaluating the use of SDF measurement through TUNEL using flow cytometry (a direct and objective assay) to predict IUI outcome. Moreover, the assay was consequently performed on fresh semen samples, limiting the possible interference of freezing and thawing on the SDF results [38,39].

We mirrored the GEE model used by Thijssen et al. [40], as we agree that the statistical model should consider the correlation between observations from the same person/couple, which logistic regression fails to do. We found that approximately one in three couples with mild male infertility or idiopathic infertility have a clinical pregnancy, and roughly one in four couples reach a live birth when embarking on a natural cycle IUI treatment, with a maximum of four IUI cycles and no multiples reported. An overall CPR of 14.7% and LBR of 10.7% per cycle was observed. This is in line with, or slightly higher than, prospective cohort studies looking into IUI outcomes [40,41]. 

We found no statistically significant differences between patients who reached CP/LB or not with regard to SDF measured at the time of IUI treatment or at diagnosis. The latter is in contrast with findings from Rex et al., who reported a significant difference when applying a baseline DNA fragmentation index (DFI) cut-off of 10% with a CPR of 25.5% (DFI ≤ 10) versus 6.1% (DFI > 10) in non-stimulated cycles of [41]. A note of caution is necessary, however, when interpreting these results, as the study applied the cut-off of 10% based on logistic regression without considering multiple observations from the same couple, and the sample size was not powered for this sub-analysis; respectively, only 47 and 82 cycles were included. A recent large, albeit retrospective, study by Zhu et al. looking at 1500 IUI cycles found no statistical difference between the low and high DFI group in pregnancy outcome [42].

The most interesting finding is the inverse relationship found between SDF in the diagnostic sample and CP/LB. This is consistent with the literature, in which elevated SDF is associated with lower pregnancy/LB rates, albeit in vivo/in vitro [4,43,44]. Surprisingly, we did not find a similar significant association for the SDF values of the respective IUI samples. Nonetheless, taking confounders into account, the models did show a trend towards significance, with *p*-values close to 0.10 or below 0.10, possibly due to the limited numbers of CP/LB. This discrepancy could be attributed to the fact that part of the inseminate had to be used for SDF testing, while during diagnosis the entire sample is available for diagnosis. In addition, we prioritized IUI treatment above performing SDF measurement at all costs. It may be the case that men with low motile sperm counts in the IUI sample had high SDF values at that time; however, these samples were maximally utilized for insemination and were not analyzed for SDF. Furthermore, because this was a “real life” setting, the time elapsed between diagnosis and insemination was different among the couples (median 93.5 days). Variations in SDF for intervals >100 days between diagnosis and insemination might explain the discrepancy in reaching significant findings for the IUI sample and treatment outcome. With regard to SDF stability over time, we refer to the paper by Punjabi et al. [45]. The authors observed that there is quite a lot of variation in SDF parameters; even within individuals with mild/unexplained male infertility and together with the high and low SDF groups, there are one out of three men who fluctuate between the two. Smit et al. hypothesized that in patients with a more severe disturbance in spermatogenesis, the chromatin structure is so compromised that the deleterious effect of unknown factors causing fluctuations in sperm DNA damage may not be as profound as in men with normal spermatogenesis and consequent normozoospermia and normal endocrine levels [46].

Female age is inversely related to clinical pregnancy, as was found in the generalized equation estimations by Thijssen et al. [40]; contrary to expectations, however, this was not the case for live birth in this study group. This inconsistency may be due to the lower number of cases (LB n = 32 versus CP n = 41) in a relatively homogenous female population with predetermined age limits. Another surprising finding is the positive relationship between reported mean cycle length and LB. Considering that we found no significant effect of natural cycle versus clomiphene citrate-treated cycles, it may be plausible that patients with longer menstrual cycles simply benefitted from cycle monitoring in timing the fertile window. 

Several authors have proposed SDF cut-offs for the prediction of treatment outcome, the treatment being IUI and IVF of ICSI [6,25,37]. This study did not distinguish a clinically significant SDF cut-off for live birth. The ROC analysis proposed 17.5% as the optimal cut-off; unfortunately, the low AUC indicates a very low discriminating power of total SDF in the ejaculate. On the premise of no LB being seen when SDF diagnosis ≥ 17.5% (an NPV of 100%), one would be keen to welcome and implement this cut-off in the clinical decision making, and we believe that if SDF diagnosis values are above 17.5% this could be used as an indicator of whether or not to use IUI as a treatment strategy. However, in view of the low positive predictive value, we believe it is important to bear in mind that low SDF does not guarantee more pregnancies [12]. 

Perhaps a more holistic approach might be warranted, including examining different male characteristics instead of focusing on just one parameter. This seems to be reflected by the predicted probabilities model that included the SDF, BMI, and smoking status of the male, which yields a higher AUC of 0.68. This combination of findings provides support for the conceptual premise that SDF might have a place as an add-on test in the diagnostic phase.

The strengths of the present study lie in its prospective nature and use of a well-defined study population in order to limit the impact of important confounders. The age limitation for women was based on the known steep decline in pregnancy outcome above 40 years old, while the more liberal upper limit for male age served to facilitate inclusion of couples despite a possible age gap and to avoid a priori exclusion of men with possibly elevated SDF. Another strength is the statistical preparation and transparency of the study, in contrast to most papers on the subject, which neglect or fail to disclose an a priori power analysis [8].

Furthermore, performing the TUNEL assay with flow cytometry, a direct and objective test, is another important advantage, as is the opportunity to perform the testing in both the diagnostic and therapeutic phases. As mentioned above, certain cycles were converted to ovulation induction due to long follicular phases during cycle monitoring. Another limitation of this study is that we did not perform an SDF test at every possible event, either due to planning errors, or more often due to insufficient semen count to perform IUI. A final possible limitation is the sample size, as the number of outcomes did not allow us to build a model with many variables included at once.

Overall, this study strengthens the hypothesis of an inverse relationship between SDF and CP/LB. Furthermore, taken together with other clinical characteristics, SDF might provide more insight into male reproductive potential and in predicting IUI outcome, although it fails to do so as an independent predictor. We believe these results have added value in counseling couples with unexplained or mild male infertility who are contemplating IUI treatment, as they highlight that in addition to female age, other factors (SDF, BMI, smoking status), including modifiable ones, might impact their treatment outcome. Further research is necessary to establish the diagnostic and prognostic efficiency of SDF as an add-on test. 

## Figures and Tables

**Table 1:** Baseline patient characteristics.

	(*n*)		Mean (SD)	
Age women (y)	114		30.3	(3.7)
Age men (y)	114		32.8	(5.3)
BMI women	102		23.7	(3.7)
BMI men	86		25.3	(3.5)
Smoking status women *	113		8	(7.1%)
Smoking status men *	112		23	(20.5%)
Infertility status women *	114	primary	78	(68.4%)
		secondary	36	(31.6%)
Infertility status men *	114	primary	81	(71.1%)
		secondary	33	(28.9%)
Infertility duration (months)	114		23.3	(12.4)
Recurrent miscarriage *	114		8	(7%)
Mean cycle length (days)	111		29	(2.4)
Tubal patency *	111	bilateral	101	(91%)
		unilateral	10	(9.1%)
Endometriosis I, II *	113		13	(11.5%)
Basal FSH (IU/L)	73		7.2	(2.5)
AMH (mcg/L)	84		3.1	(2.4)
Hunault score (%)	113		18.1	(5.5)

* *n* (%) for categorical variables.

**Table 2:** Diagnostic semen analysis.

	(*n*)	Median	[IQR]
Abstinence (d)	112	4	[2]
Concentration (M/mL)	112	48	[63.7]
Volume (mL)	112	3.7	[2.3]
Total count (M)	113	165.2	[199]
Progressive motility (%)	112	55	[16]
TPMSC	112	88.8	[117.1]
Morphology (%)	112	5	[5]
Leukospermia (n (%))	112	4	[3.5]
% SDF total in ejaculate	104	9	[7.2]
% SDF vital in ejaculate	104	1	[0.9]
% SDF total in gradient	102	9.6	[15.5]
% SDF vital in gradient	102	0.6	[0.8]

TPMSC: total progressive motile sperm count.

**Table 3:** IUI characteristics.

*IUI Cycle*					*IUI Semen Analysis*			
	(*n*)					(*n*)	Median	[IQR]
Stimulation protocol	301	NC	278	(92.4%)	Abstinence (days)	299	4	[2]
		Cc	23	(7.6%)	Volume (mL)	299	3.7	[2.4]
Ovulation trigger	302	HCG admin	234	(77.5%)	Concentration (M/mL)	299	43.1	[47.8]
		LH surge	68	(22.5%)	Total sperm count (M)	299	148.6	[167]
Estradiol (ng/mL)	302		249	(92)	Progressive motility (%)	299	45	[20]
Progesterone	302		0.3	(0.2)	IMC (M)	299	4.7	[5]
Cycle day ovulation trigger	302		13	(3.5)	% SDF total in ejaculate	209	8	[7]
Number of dominant follicles	302	1	287	(95.0%)	% SDF vital in ejaculate	209	1	[2]
		2	15	(5.0%)	% SDF total in gradient	210	8.3	[10]
Endometrial thickness (mm)	298		9.2	(2)	% SDF vital in gradient	210	0.4	[0.8]

(*n*) = number of valid observations; mean (SD) for the continuous variables; *n* (%) for the categorical variables; median [IQR]; NC = Natural cycle; Cc = clomiphene citrate; IMC = inseminating progressive motile count. *n* = 1 received gonadotrophin stimulation in cycle 4.

**Table 4:** GEE model for the odds of clinical pregnancy (univariate analysis).

	OR	[Lower,Upper CL]	*p* Value		OR	[Lower,Upper CL]	*p* Value
**Patient**				**IUI cycle**			
Age women (y)	0.92	[0.85, 0.99]	**0.03**	Treatment protocol (clomid/natural cycle)	1.31	[0.39, 4.38]	0.66
BMI women	1.07	[0.98, 1.16]	0.14				
Smoking status women (yes/no)	0.53	[0.13, 2.24]	0.39	Size dominant follicle (mm)	0.85	[0.70, 1.03]	0.09
Age men (y)	0.95	[0.89, 1.01]	0.11	Size dominant follicle categorical(≥17/<17 mm)	0.49	[0.14, 1.64]	0.24
BMI men	0.93	[0.85, 1.02]	0.14				
Smoking status men (yes/no)	0.69	[0.31, 1.55]	0.37	Number of dominant follicles (2/1)	1.43	[0.39, 5.28]	0.59
Previous fertility treatment (yes/no)	1.12	[0.49, 2.60]	0.78				
Tubal Patency (Unilateral/bilateral)	0.46	[0.10, 2.14]	0.32	Ovulation trigger (HCG-administration/ spontaneous LH surge)	1.30	[0.58, 2.91]	0.52
Endometriosis I, II (yes/no)	0.32	[0.07, 1.52]	0.15				
Basal FSH (IU/L)	0.94	[0.79, 1.11]	0.45				
AMH (mcg/L)	0.97	[0.82, 1.14]	0.67	Estradiol day of ovulation trigger (≥200 versus <200 ng/L)	0.77	[0.39, 1.53]	0.46
Reported mean cycle length (d)	1.05	[0.92, 1.21]	0.46				
Infertility duration (y)	0.97	[0.93, 1.01]	0.13	Endometrial thickness day of ovulation trigger (≥7 versus <7 mm)	1.08	[0.35, 3.35]	0.89
Infertility classification (primary/secondary)	1.57	[0.77, 3.19]	0.21				
**Diagnosis semen analysis**				**IUI semen analysis**			
Abstinence (d)	0.95	[0.85, 1.06]	0.36	Abstinence (d)	1.05	[0.93, 1.19]	0.44
Volume (ml)	0.97	[0.80, 1.18]	0.77	Volume (ml)	1.09	[0.90, 1.31]	0.38
Concentration (M/mL)	0.99	[0.99, 1.00]	0.09	Concentration (M/mL)	1.00	[0.99, 1.01]	0.35
Grade A+B motility (%)	1.00	[0.97, 1.03]	0.99	Grade A+B motility (%)	1.00	[0.98, 1.02]	0.65
Ideal morphology (%)	0.98	[0.89, 1.08]	0.65	IMC (M)	0.99	[0.91, 1.07]	0.76
SDF total in ejaculate (%)	0.94	[0.90, 0.99]	**0.02**	SDF total in ejaculate (%)	0.97	[0.92, 1.02]	0.25
SDF vital in ejaculate (%)	0.89	[0.64, 1.23]	0.48	SDF vital in ejaculate (%)	1.11	[0.94, 1.32]	0.21
SDF total in gradient (%)	1.00	[0.98, 1.03]	0.91	SDF total in gradient (%)	0.97	[0.94, 1.01]	0.12
SDF vital in gradient (%)	1.12	[0.75, 1.67]	0.57	SDF vital in gradient (%)	1.18	[0.76, 1.83]	0.47

**Table 5:** GEE model for the odds of live birth (univariate analysis).

	OR	[Lower,UpperCL]	*p* Value		OR	[Lower,UpperCL]	*p* Value
**Patient**				**IUI cycle**			
Age women (y)	0.93	[0.85, 1.02]	0.11	Treatment protocol (clomid/natural cycle)	1.88	[0.56, 6.33]	0.31
BMI women	1.04	[0.94, 1.15]	0.49				
Smoking status women (yes/no)	/	/	/	Size dominant follicle (mm)	0.90	[0.73, 1.10]	0.31
Age men (y)	0.96	[0.90, 1.02]	0.21	Size dominant follicle categorical (≥17/<17 mm)	0.47	[0.15, 1.46]	0.19
BMI men	0.93	[0.84, 1.03]	0.15				
Smoking status men (yes/no)	0.48	[0.17, 1.38]	0.17	Number of dominant follicles (2/1)	2.03	[0.54, 7.55]	0.29
Previous fertility treatment (yes/no)	1.04	[0.38, 2.83]	0.94				
Tubal Patency (Unilateral/bilateral)	0.65	[0.14, 3.05]	0.58	Ovulation trigger (HCG-administration/ spontaneous LH surge)	1.06	[0.44, 2.56]	0.89
Endometriosis I, II (yes/no)	0.21	[0.03, 1.70]	0.14				
Basal FSH (IU/L)	0.93	[0.78, 1.11]	0.40				
AMH (mcg/L)	1.03	[0.87, 1.22]	0.72	Estradiol day of ovulation trigger (≥200 versus <200 ng/L)	0.97	[0.44, 2.16]	0.95
Reported mean cycle length (d)	1.15	[1.00, 1.32]	0.06				
Infertility duration (y)	0.94	[0.88, 0.99]	**0.03**	Endometrial thickness day of ovulation trigger (≥7 versus <7 mm)	1.10	[0.30, 4.04]	0.88
Infertility classification (primary/secondary)	1.51	[0.69, 3.27]	0.30				
**Diagnosis semen analysis**				**IUI semen analysis**			
Abstinence (d)	0.95	[0.84, 1.08]	0.45	Abstinence (d)	1.05	[0.92, 1.20]	0.45
Volume (ml)	0.92	[0.73, 1.15]	0.47	Volume (ml)	0.96	[0.78, 1.17]	0.66
Concentration (M/mL)	1.00	[0.99, 1.00]	0.37	Concentration (M/mL)	1.00	[0.99, 1.01]	0.94
Grade A+B motility (%)	1.00	[0.97, 1.03]	0.87	Grade A+B motility (%)	1.01	[0.99, 1.03]	0.52
Ideal morphology (%)	1.00	[0.89, 1.12]	0.97	IMC (M)	1.03	[0.95, 1.11]	0.53
SDF total in ejaculate (%)	0.95	[0.90, 1.00]	**0.04**	SDF total in ejaculate (%)	0.96	[0.90, 1.02]	0.17
SDF vital in ejaculate (%)	0.89	[0.59, 1.34]	0.58	SDF vital in ejaculate (%)	1.07	[0.94, 1.23]	0.31
SDF total in gradient (%)	1.00	[0.98, 1.03]	0.84	SDF total in gradient (%)	0.96	[0.91, 1.01]	0.10
SDF vital in gradient (%)	1.09	[0.71, 1.69]	0.69	SDF vital in gradient (%)	1.18	[0.73, 1.92]	0.50

## Data Availability

The data presented in this study are available from the corresponding author upon reasonable request.
